# Atlanta Medical College Clinic

**Published:** 1878-01

**Authors:** S. W. Johnson


### ATLANTA MEDICAL COLLEGE CLINIC—SERVICE OF PROF. J. G. WESTMORELAND.

Reported by S. W. Johnson.

November 5, 1877.—M. B., colored, male, æt 60, by occupation, whitewasher.

Gentlemen: The history of this patient, as just given by himself, is duly considered in connection with existing symptoms. You remember his statement, that for four or five weeks during last summer he was confined to bed with painful swellling and soreness of the ankles and feet. That description answers well for acute rheumatic inflammation. His present symptoms denote subacute inflammation of the same character in the right knee and ankle. While implicit confidence cannot, of course, be had in the details of such histories, they are important, and sometimes indispensable factors, in the formation of correct diagnosis. Some constitutions are prone to rheumatism, and the history giving evidence of a former attack confirms us in the opinion to which the symptoms now present has led. The soreness, imperfect inability and slight pain now present are perhaps due in part to the results of the former attack, and to a return of the disease in a subacute form. It is not uncommon to find stiffness and even soreness of the joints following acute rheumatism, probably caused by deposits on the ligaments and other parts connected with the articulation. These symptoms are particularly noticeable in cloudy weather, and are probably then aggravated by electrical changes.

In a case purely the result of rheumatic inflammation, without any return of the disease, the proper treatment for rheumatism proper will not be found successful. This condition of the joints following rheumatism and the painful soreness along the course of nervous trunks, and aggravated by stormy weather, are called chronic rheumatism, and are probably both caused by the deposit above mentioned. This condition is more rationally treated with means calculated to produce catalysis of the deposited and semi-organized fibrin. Preparations of iodine, particularly that of iodide potassium, have been used for this purpose: Muriate of ammonia will also probably be found useful.

Finding in the case before us evidences of actual rheumatism, I shall more particularly allude to this condition now. Rheumatism consists of a peculiar inflammation affecting more frequently fibrous structures. While some of the local and general symptoms are identical with those of ordinary inflammation, others differ materially. Swelling, soreness, pain, and general fever, exist as in traumatic or other cause, but the migratory character and result on the diseased part, make rheumatism a special inflammation. In uncomplicated rheumatism, suppuration never occurs, and the painful swelling and redness disappear from a joint in a few hours, attacking another in a similar manner.

These strange features of the disease bring us to the consideration of its patholgy. It has been supposed that morbific material, of particular kind, circulating in the blood, causes the local disturbance in question. A more reasonable conclusion, however, I think, is that primary disturbance of the spinal nervous system leads to the migratory manifestations of rheumatism. The exact condition of the cord which causes such development in the joints is not well understood, but the sudden transition from one part to another is conclusive against the theory of deposit from the blood, causing the local inflammation; to say nothing of the deductions to be drawn from the succesful mode of treatment. Those who advocate the blood theory use different and exactly opposite means of cure. One uses alkalies to neutralize a supposed acid in the blood, and the patient recovers; another gives lemon drinks and other acids to destroy the alleged alkaline deposit in the joints, and his patients finally recover. Now the explanation of the recoveries after the use of these opposite plans of treatment, is that the disease will abate without any treatment, as in the case before us. In four or five weeks he recovered from the attack last summer, and, doubtless, without any beneficial treatment.

I adopted a treatment directed to the spinal cord many years ago, and without any settled pathological views on the subject. I read an article in some medical journal, giving the treatment of rheumatism by quinine and opium, which I adopted the first opportunity, knowing no injury could result to the patient. Finding, as I supposed and still believe, benefit from these remedies, I have continued them as the basis of my treatment since, and as proof of the pathological condition leading to the local symptoms.

It is known that quinine is a spinal tonic, from results obtained under various circumstances. Again, in cases which do not readily yield to opium and quinine, I find, often, prompt relief after blistering over the spinal column. Recently, much has been said in journals about the curative action of salisylic acid in rheumatism, and, if true, is corroborative of my theory of its pathology, and also of the propriety of the quinine treatment. It is generally known that salicin is a spinal tonic, having about the same action of quinine, and as salicylic acid is the product of salicin, it is reasonable to suppose that it will answer the purposes of quinine. I have made one test of the acid in rheumatism, and, although one trial is not sufficient to decide upon its merits, I do not think it superior to quinine. After using one hundred and twenty grains in the case during twenty-four hours, I became anxious to effect a cure, and resorted to full doses of opium and quinine, say two grains of the former ($\frac{1}{3}$ gr. morphine) and ten of the latter, repeated every six hours until three or four doses had been taken, at the same time having the spine thoroughly blistered. In thirty-six hours complete relief and rapid convalescence followed.

In the case before us I prescribe:

℞—Quinine, . . . . . . grs. xxx
Morphine, . . . . . . . gr. j

Mix and make three powders.

Sig.—One at night, morning and noon, in syrup.

A blister two by ten inches applied to the lumbar and sacral spine.

